# Current usage of and perspectives on the use of MRI to assess treatment non-response in axial spondyloarthritis: a UK-based survey

**DOI:** 10.1093/rap/rkae139

**Published:** 2024-11-28

**Authors:** Jake Weddell, Timothy J P Bray, Raj Sengupta, Dennis McGonagle, Pedro M Machado, Helena Marzo-Ortega

**Affiliations:** NIHR Leeds Biomedical Research Centre, Leeds Teaching Hospitals Trust, Leeds, UK; Leeds Institute of Rheumatic and Musculoskeletal Medicine, University of Leeds, Leeds, UK; Centre for Medical Imaging, University College London, London, UK; Department of Imaging, University College London Hospital, London, UK; Royal National Hospital for Rheumatic Diseases, Royal United Hospitals NHS Foundation Trust, and University of Bath, Bath, UK; NIHR Leeds Biomedical Research Centre, Leeds Teaching Hospitals Trust, Leeds, UK; Leeds Institute of Rheumatic and Musculoskeletal Medicine, University of Leeds, Leeds, UK; Department of Neuromuscular Diseases and Centre for Rheumatology, University College London, London, UK; NIHR University College London Hospitals Biomedical Research Centre, University College London Hospitals NHS Foundation Trust, London, UK; Department of Rheumatology, Northwick Park Hospital, London North West University Healthcare NHS Trust, London, UK; NIHR Leeds Biomedical Research Centre, Leeds Teaching Hospitals Trust, Leeds, UK; Leeds Institute of Rheumatic and Musculoskeletal Medicine, University of Leeds, Leeds, UK

Key messages

- Research is needed on the role of MRI to assess disease activity and treatment non-response in axSpA.

Dear Editor, Clinical assessment of response to biologic or targeted synthetic DMARDs in axial spondyloarthritis (axSpA) can be challenging due to the lack of specific biomarkers and several non-inflammatory pathologies potentially contributing to patient symptoms of pain and stiffness. MRI is routinely used to identify active inflammation that can aid the diagnosis of axSpA. Whereas current National Institute for Health and Care Excellence guidance has no specific imaging recommendations on disease monitoring in axSpA [1], the most recent ASAS/EULAR guidance states that ‘MRI is not routinely recommended for monitoring, as its value for this purpose is still unclear and its frequent use is considered unfeasible due to the high costs’ [2].

Recent data suggest a potential disconnect between response in objective measures of inflammation such as MRI and CRP and clinical outcome responses in patients with axSpA, highlighting the risk of underestimating anti-inflammatory treatment effects when relying on patient-reported outcomes alone [3]. This is also a common scenario at the bedside, where clinicians often struggle to understand treatment response based solely on clinical symptoms of pain, fatigue and stiffness, which may flare or persist despite adequate suppression of CRP levels or BASDAI improvement, thus suggesting an important role for imaging to assess objective response to treatment and guide decision-making.

A recent clinical audit reported in the Leeds Specialist Spondyloarthritis Service, a large tertiary centre, identified that 16.7% of MRIs performed under the axSpA or inflammatory back pain protocol were requested to complement assessment of disease activity in patients reporting a loss of response to their current biologic therapy, with findings influencing the treatment decision in half of the patients scanned [4]. These data highlight the importance of MRI in pragmatic clinical practice. In order to assess current practice and perspectives on the use of MRI to assess treatment non-response in axSpA among UK rheumatologists, we designed an online survey consisting of 10 questions (see Supplementary Data S1, available at *Rheumatology Advances in Practice* online). The survey was sent to rheumatologists through the British Society for Spondyloarthritis (BRITSpA) and dedicated email channels between 11 May and 26 June 2024. No ethical approval was required for this voluntary survey of a professional group.

A total of 77 replies were received from UK-based rheumatologists. All respondents (100%) reported performing MRI to assess treatment non-response in their practice, although only a minority did so routinely [*n* = 8 (10.3%)], with 28 (36.4%) reporting frequent use, 38 (49.4%) infrequent use and 3 (3.9%) rare use. However, slightly less than half of respondents (46.7%) reported an increase in the use of MRI to assess non-response in recent years. In addition, and despite the lack of confirmatory data, the vast majority asked patients to stop their NSAIDs prior to MRI (83.1%); however, as expected, there was limited consensus on the length of time to withhold NSAIDs, with responses ranging from 48 h to 14 days. The majority of UK respondents reported scanning the whole spine and sacroiliac joints (74.0%), in accordance with the BRITSpA consensus guidelines [5]. All respondents felt that there was a role for MRI in the assessment of treatment non-response, with 16.9% stating that MRI should be performed in all cases. Nevertheless, 62.3% and 63.6% mentioned cost implications and MRI waiting time, respectively, as barriers to routine MRI use. Indeed, a recent freedom of information report outlined new challenges aggravated by the recent COVID-19 pandemic, including the increase in waiting times for MRI, reliance on outsourcing and the reporting of MRI by non-musculoskeletal radiologists [6], which today have a clear impact in the utilization of MRI in the National Health Service. Yet, and although our current survey numbers may appear small, they are representative of the limited number of rheumatologists who work in the field of SpA in the UK, with the majority in general hospitals with limited resources and no dedicated SpA clinics. As such, these data give a true snapshot of current practice and suggest the need for further research in this area in order to identify the role of MRI in disease monitoring in axSpA.

In conclusion, the use of MRI to assess non-response to treatment with advanced therapies in axSpA appears to be common in the UK, despite the lack of supporting evidence and contrary advice from the most recent ASAS/EULAR guidelines. Although there were wide variations in practice among respondents, and in the context of a disease with no specific biomarkers of treatment response, there was unanimous agreement on a role for MRI in assessing this patient group. Given the reported increased use of MRI in recent years and concerns about cost implications, further research to identify which patients would most benefit and the cost-effectiveness of this strategy is required.

## Supplementary Material

rkae139_Supplementary_Data

## Data Availability

All data relevant to this study are included in the article or uploaded as online supplemental information.
