# Supplementary material for: Current usage of and perspectives on the use of MRI to assess treatment non-response in axial spondyloarthritis: a UK-based survey
Source: Rheumatol Adv Pract. 2024 Nov 28;8(4):rkae139. doi: 10.1093/rap/rkae139 (PMC11645469; doi:10.1093/rap/rkae139)
Supplement: rkae139_Supplementary_Data [file rkae139_supplementary_data.docx]

**Supplementary materials**

**Supplementary Data S1- Questions and answer choices from survey sent to clinicians.**

Q1 How often do you perform MRI of the spine and/or sacroiliac joints in patients experiencing treatment non-response in your clinical practice?

Routinely/ all of the time

Frequently/ most of the time

Infrequently/ some of the time

Rarely

Never

Q2 Do you believe there is a role for MRI in assessing treatment non-response in axSpA?

Yes- in all patients

Yes – in some patients

No

Q3 Please elaborate on your answer to Q2 (freetext)

Q4 How has your usage of MRI to assess treatment non-response changed in the last few years?

Increased

Stayed the same

Decreased

Not sure

N/A

Q5 Which anatomical region do you request an MRI of in patients experiencing non-response?

Whole spine + SIJ

Lumbar spine + SIJ

SIJ only

Symptomatic areas only

I would not request an MRI to assess treatment non-response

Q6 Which of the following would make it more likely for you to request an MRI to assess treatment non-response (tick all that apply)

Primary non-response / lack of response

Secondary non-response / lack of response

Non-response to multiple biologic/ targeted synthetic DMARD treatment

To exclude non-inflammatory causes of back pain

When the patient has ongoing back pain but normal CRP

When a concomitant non-axSpA pathology is suspected

None of the above

Q7 Do you ask patients to stop NSAIDs before the MRI to assess treatment non-response?

Yes

No

Q8 If yes to question 7, how long do you ask patients to stop their NSAIDs for before the MRI? (freetext)

Q9 In your opinion, which are the barriers to using MRI to assess treatment non-response in axSpA? (tick all that apply)

Lack of evidence

Cost implications

MRI waiting times

Lack of experience interpreting MRI findings

Lack of support from experienced MSK radiologists

Other

Q10 If you answered "other" in question 9, please elaborate here (freetext)

Q11 In which country is your rheumatology practice? (freetext)
